# Therapists’ experiences of remotely delivering cognitive-behavioural or graded-exercise interventions for fatigue: a qualitative evaluation

**DOI:** 10.1093/rap/rkac083

**Published:** 2022-10-17

**Authors:** Sarah E Bennett, Celia Almeida, Eva-Maria Bachmair, Stuart R Gray, Karina Lovell, Lorna Paul, Alison Wearden, Gary J Macfarlane, Neil Basu, Emma Dures

**Affiliations:** Musculoskeletal Research Unit, Bristol Medical School, University of Bristol, Bristol, UK; School of Health and Social Wellbeing, University of the West of England, Bristol, UK; Academic Rheumatology, Bristol Royal Infirmary, Bristol, UK; Aberdeen Centre for Arthritis and Musculoskeletal Health (Epidemiology Group), University of Aberdeen, Aberdeen, UK; Institute of Cardiovascular and Medical Sciences, University of Glasgow, Glasgow, UK; Division of Nursing, Midwifery & Social Work, University of Manchester, Manchester, UK; School of Health and Life Science, Glasgow Caledonian University, Glasgow, UK; Division of Psychology and Mental Health, University of Manchester, Manchester, UK; Aberdeen Centre for Arthritis and Musculoskeletal Health (Epidemiology Group), University of Aberdeen, Aberdeen, UK; Institute of Infection, Immunity and Inflammation, University of Glasgow, Glasgow, UK; School of Health and Social Wellbeing, University of the West of England, Bristol, UK; Academic Rheumatology, Bristol Royal Infirmary, Bristol, UK

**Keywords:** fatigue, qualitative, exercise, cognitive-behavioural approaches, rheumatic diseases

## Abstract

**Objective:**

Fatigue is a challenging feature of all inflammatory rheumatic diseases. LIFT (Lessening the Impact of Fatigue in inflammatory rheumatic diseases: a randomized Trial) included remotely delivered personalized exercise programme (PEP) or cognitive-behavioural approach (CBA) interventions. The aim of this nested qualitative evaluation was to understand rheumatology health professionals’ (therapists’) perspectives of delivering the interventions in the LIFT trial.

**Methods:**

A subgroup of therapists who had delivered the personalized exercise programme (PEP) and cognitive-behavioural approach (CBA) interventions took part in semi-structured telephone interviews.

**Results:**

Seventeen therapists (13 women and 4 men) who delivered PEP (*n* = 8) or CBA (*n* = 9) interventions participated. Five themes were identified. In ‘The benefits of informative, structured training’, therapists described how they were able to practice their skills, and the convenience of having the LIFT manual for reference. When ‘Getting into the swing of it’, supporting patients gave therapists the confidence to tailor the content of the manual to each patient. Clinical supervision supported therapists to gain feedback and request assistance when required. In ‘Delivering the intervention’, therapists reported that patients valued the opportunity to talk about their fatigue and challenge their beliefs. In ‘Challenges in delivering the LIFT intervention’, therapists struggled to work in partnership with patients who lacked motivation or stopped engaging. Finally, in ‘LIFT developing clinical skills’, therapists gained confidence and professional satisfaction, seeing patients’ fatigue improve over time.

**Conclusion:**

The findings support the provision of training for rheumatology health professionals to remotely deliver fatigue-management interventions. Insights from these trials can be used to better improve clinical practice and service provision.

Key messages

- Skills training for rheumatology health professionals can be used successfully to deliver fatigue-management interventions remotely.
- Therapists described increased professional satisfaction and confidence when seeing patients’ fatigue improve.
- These insights inform strategies for service provision and clinical practice for remotely delivered support.

## Introduction

Fatigue can be an overwhelming and distressing feature of inflammatory rheumatic diseases (IRDs). Most of the evidence to date has come from studies in RA, which have established that between 42 and 80% of patients experience significant fatigue, which they can find difficult to manage [1–3]. Similar findings have been reported for other IRDs, including SLE [4] and AS [5–7].

A qualitative metasynthesis found that patients often experience fatigue as an unpredictable and pervasive symptom with physical, cognitive, emotional and social effects [8]. The authors concluded that it is important for health professionals to acknowledge the impact of fatigue on the everyday lives of patients and provide support to develop strategies to cope well, increase physical activity and maintain work [8]. This is consistent with a systematic review of non-pharmacological interventions that found evidence to support psychosocial and physical activity interventions [9].

Although cognitive-behaviourally based approaches have been used widely within psychology, a growing need for non-psychologically trained health-care professionals to deliver psychologically informed care has been recognized [10–12]. There are a number of examples within the literature of health-care professionals being trained in new, psychologically informed skills, such as cognitive-behavioural approach (CBA) training, including CBA interventions for low back pain, delivered by trained nurses in primary care [13]. Likewise, the RAFT (Reducing Arthritis Fatigue- clinical Teams) trial, a seven-session group course for people with RA-related fatigue, was delivered by trained rheumatology health-care professionals (occupational therapists and nurses) using cognitive-behavioural principles [14]. Given that access to clinical psychology within rheumatology teams is not always available and can be difficult for patients to access [14], if health-care professionals could be trained to deliver an effective CBA intervention, this could potentially offer benefit to patients with IRD-related fatigue.

Lessening the Impact of Fatigue in inflammatory rheumatic diseases: a randomized Trial (LIFT) is a multicentre, three-arm randomized trial using a remotely delivered personalized exercise programme (PEP) or CBA intervention, in addition to usual care (a Versus Arthritis patient information leaflet) [15]. Further detail about the LIFT intervention has been published separately [16]. The interventions were designed to facilitate cognitive and behavioural change, to enhance patients’ coping and self-management and to reduce the severity and impact of their fatigue. The intervention was delivered by health professionals (termed therapists in this article), who were members of National Health Service (NHS) staff at each research site; CBA by a rheumatology nurse or equivalent allied health professional, such as an occupational therapist, and PEP by a specialist physiotherapist, usually with a background in rheumatology. Participants with IRD in the intervention arms were randomized to seven one-to-one sessions of either the PEP or CBA interventions delivered by trained therapists over 14 weeks, plus a booster session at 22 weeks. Sessions were delivered via telephone or by videoconference, depending on patient preference. In the PEP arm only, the first session was delivered face to face [15]. For the PEP intervention, participants completed detailed physical activity diaries and set personalized goals relating to what they wanted to achieve from the programme [16]. These data were used to plan a personalized progressive exercise programme, in agreement between the therapist and participant [16]. In the CBA intervention, participants were given basic information about how cognitive, behavioural, emotional and biological factors can interact to impact fatigue. Participants were encouraged to develop a problem statement that described their own fatigue in terms of these factors and were encouraged to set goals, complete activity diaries, complete homework activities and participate in review and feedback about the intervention [16]. Progress was reviewed in each session, and new goals were put in place if required [16].

Therapists delivered the LIFT intervention after separate PEP and CBA training sessions. Initial training for PEP was 2 days, with additional training for new therapists reduced to 4–5 h on a single day. Initial training for CBA was 3 days, with additional training for new therapists reduced to 2 days. In both PEP and CBA training, more efficient and shorter training was used for subsequent sessions. Training was delivered face to face by experienced clinical academics (A.W., K.L. and L.P.) and featured vignettes of fatigue cases, role play and skills practice [15]. During the period when they were delivering the interventions to trial participants, therapists had access to clinical supervision every 2 weeks or as needed. Clinical supervision was provided by A.W., K.L., S.R.G. or L.P. via telephone [15]. The aim of this study was to evaluate therapists’ experiences of intervention training and delivery as part of the LIFT trial.

## Methods

We used qualitative methods and collected data in semi-structured telephone interviews with a subgroup of LIFT therapists who had delivered the PEP and CBA interventions in the LIFT randomized controlled trial. Qualitative methods are well suited to in-depth exploration of topics [17, 18]. The interview schedules for the PEP and CBA arms are outlined in Supplementary Data S1, available at *Rheumatology Advances in Practice* online, and featured open-ended questions designed by the study team. Questions explored therapists’ reasons for taking part in LIFT, prior relevant experience, thoughts on the training and delivery, impact on the therapists’ clinical practice and any suggestions for changing the intervention for future roll out.

### Sample

All therapists who had delivered LIFT intervention sessions at the six participating NHS sites were eligible to take part. The LIFT therapists were sent invitations to take part in the nested qualitative evaluation sub-study (*n* = 27) after they had completed their delivery as part of the trial. Therapists returned reply slips to the first author (S.E.B.) to express an interest in taking part. All therapists provided written informed consent for the qualitative component. To maintain anonymity, participant codes have been used throughout.

### Data collection

Interviews were conducted by C.A. and S.E.B., research associates with prior experience of conducting telephone interviews but with no involvement in the design or delivery of the LIFT training or interventions. Before the start of each interview, therapists were reminded that the call was being recorded, the procedure for anonymization and the aims of the interview, and they were given the opportunity to ask any questions.

### Data analysis

Audio recordings were transcribed by an approved transcription service, anonymized, and checked for accuracy against the original audio recordings. The transcripts were imported into NVivo 12 (released in 2018) [19] and analysed using inductive thematic analysis as outlined by Braun & Clarke [20], a data-driven approach, with no overarching framework applied to the data *a priori*. The underpinning perspective was realist, with analysis at the latent level. The first author (S.E.B.) read through all the transcripts and coded text that related to the research questions. Codes were reviewed, revised and organized into overarching themes and subthemes, with some codes raised and upgraded into themes, while less relevant codes were discarded [20]. Data saturation was determined when no new themes were identified from therapist interviews [21]. Two transcripts were reviewed independently by four co-authors (E.D., C.A., A.W. and K.L.) and the themes and subthemes discussed as a team to reach consensus. Themes and subthemes identified in the thematic analysis can be seen in Table 1.

**Table 1.** Themes and subthemes identified in the thematic analysis

Theme	Subtheme
1. The benefits of informative, structured training	Mixing it up (benefits of training)
	A lot to take in at once
	Nervous, but keen to try
2. Getting into the swing of it	Therapist utilization of the training manual
	Supervision gives ‘input from a different angle’
3: Delivering the intervention	Building rapport
	More open communication
4. Challenges in delivering the LIFT intervention	Patients unable or unwilling to engage
	Patients underestimating the work required
5. LIFT developing clinical skills	Professional satisfaction
	Implementing the LIFT intervention in daily practice
	An intervention that still works with COVID

COVID: coronavirus disease; LIFT: Lessening the Impact of Fatigue in inflammatory rheumatic diseases: a randomized Trial.

### Ethics

The study complied with the Declaration of Helsinki and was approved by the Wales Research Ethics Committee Number 7 (reference: 17/WA/0065). Informed consent was obtained from all participants.

## Results

A total of 17 therapists (13 women and 4 men) from the PEP (*n* = 8) and CBA (*n* = 9) arms responded and were able to participate in telephone interviews. Interviews were conducted between July 2019 and August 2020. Therapists who did not respond to invitations to participate were not interviewed; therefore, any reasons for non-participation in the interviews were not recorded. The 17 therapists who were interviewed had attended one of four training sessions: 3-day in-person training (*n* = 6), 2-day in-person training (*n* = 5), 4-h intensive in-person training (*n* = 3) or 4-h intensive remotely delivered training (*n* = 3). This reflects the health professionals joining the LIFT study at different time points. Interviews lasted between 25 and 45 min (average 34 min).

### The benefits of informative, structured training

Therapists valued the ability to train with other rheumatology health professionals before delivering the intervention. Many identified the benefits of having informative and structured training to guide them in delivery.

#### Mixing it up (benefits of training)

Although role play was not everyone’s ‘favourite thing’ [T02 CBA], a variety of methods helped therapists to practise their skills before meeting patients. Therapists approved of the variety in the content and delivery of their training, because it enabled them to stay focused.

We weren’t sitting – they were mixing it up, they were taking turns talking, we were doing exercises and being included, so they … kept our attention right throughout the day and good breaks and things. It was ideal. I wouldn’t change a thing. [T07 CBA]

With the exercise cohort, there was a face-to-face appointment, so we did a bit of role-playing for that and a bit of role-playing for the telephone as well. [T14 PEP]

#### A lot to take in at once

This subtheme captures the challenges that therapists encountered during the training for their role.

Training times varied from 2 days to 4 h. There was a lot to absorb and learn in the longer training sessions: ‘It was … quite a lot of information to take in at one go’ [T12 PEP]. However, those in the shorter training sessions felt that they would have liked more time to practise their skills before meeting with patients.

[In 3-day training] people had opportunities to do a bit of role playing, whereas we kind of tended to gloss over that a bit because we only had 2 days … if we’d done a bit more role playing, it would have been helpful. [T05 CBA]

#### Nervous, but keen to try

Most therapists felt nervous before delivering the intervention and meeting LIFT participants for the first time, but found that they grew more confident with practice: ‘I did feel a bit anxious about that in the beginning, but actually the more I practised at it, the easier it became’ [T09 CBA]. After training, this therapist described how ‘I felt quite confident that I knew what I was doing. Certainly, once I went through my first patient from start to finish … it was confidence as you go.’ [T12 PEP].

### Getting into the swing of it

Once therapists had embarked upon the LIFT trial, they described how the chance to apply and make use of their training improved their confidence. The therapists spoke of liking the manual as a resource to refer to, alongside support from professional supervision.

#### Therapist utilization of the training manual

Although almost all therapists described being nervous at the start of delivering the interventions to participants, the chance to practise and the support provided by the manual gave them the confidence and flexibility to tailor content to individual patients’ needs and to jump back and forth between sections of the intervention.

Sometimes I use them more or less in order; sometimes I jump back and forth. No, I’ve kind of got into the swing of it now. [T03 CBA]

The better you become, the slicker you become. [T08 CBA]

Therapists gave very positive feedback regarding the intervention manual, which many liked to keep close by during sessions: ‘I could look at that while I was on the ‘phone … I actually could look at it quite confidently’. [T17 PEP].

Some therapists suggested a digital copy, both to prevent the paper-bound manual from becoming worn with regular use and to make navigating to key content easier.

I did find it difficult to use during sessions because it’s big and hard to find things, but they’re all where they should be, and it’s well designed … it’s just the nature of that much information and being able to locate it … If I had it open as a PDF, I could do a quick search. [T10 PEP].

#### Supervision gives ‘input from a different angle’

The clinical supervision provided to therapists by the LIFT trainers allowed them to query their own practice, obtain feedback on their performance and ask for input and assistance on more difficult interactions.

Trying to figure out how to apply it is a difficult thing, and that’s where the supervision was really handy, because it just comes at a different angle than I’m used to. [T03 CBA]

That gave me confidence as well.… I knew that somebody was on the end of the ’phone that could actually answer your question. [T15 PEP]

### Delivering the intervention

Therapists had the option to deliver the intervention using telephone or internet-based audio-video calls, according to patient preference. However, only the telephone option was taken up. Although many therapists had not used remote delivery before the intervention, they found telephone delivery to be straightforward.

#### Building rapport

The first face-to-face session that was part of the PEP delivery enabled therapists to build rapport with participants; ‘Because all of the participants I met one to one for their initial appointment, so I could visualize them and I knew what their capacity and things were’ [T15 PEP]*.* Likewise, although the face-to-face session was not an element of the CBA arm, therapists still enjoyed the opportunity to build a good relationship with participants: ‘Each time you feel like you get to know them a bit more and you recognize their voice … I remember you, it’s nice to speak to you again’ [T04 CBA].

#### More open communication

Therapists reported that participants were able to talk about their fatigue and seemed more open in telephone communication: ‘[LIFT] worked better because it was over the ‘phone, because there was a level of control that people had, so far as they weren’t presenting all of themselves.… It was good for them to have … a barrier that they could report and still feel independent’ [T10 PEP]. Participants could challenge their own beliefs about fatigue and the causes of their fatigue: ‘[LIFT] gave them a different view on their condition and maybe how they can look at things … they looked at things differently, and they said they had tools to carry on and manage their fatigue’ [T02 CBA], with LIFT giving them the tools to manage their fatigue better themselves.

### Challenges in delivering the LIFT intervention

#### Patients unable or unwilling

The LIFT therapists struggled to engage participants who were unable or unwilling to change their self-management behaviours, in both the PEP and CBA arms:

They tell [you] they try, but they don’t really; you can tell that it’s not really going to change. One of them said to me, ‘I know what I have to do, I’m just not really in the right frame of mind to change some habits’. [T01 CBA]

You wouldn’t necessarily feel like they’d actively changed their everyday life, which for some of them, they needed to. [T16 PEP]

#### Patients underestimating the work required

Therapists reported that a minority of participants had not realized ‘how much … work on their side they’ve got to do’ [T08 CBA] for them to obtain the best results from the LIFT interventions and see the greatest benefit. Therapists described these difficulties:

The thing that put most people off initially was the first diary and the amount of homework and being organized, and how to share that information back. [T12 PEP]

By session three you could see who were the patients that were going to try and apply all this advice and the ones that were just really not interested.… Expecting like a magic wand to come out and sort out their fatigue.… They were not quite prepared to put in the work themselves. [T01 CBA]

### LIFT helping own practice going forward

#### Professional satisfaction

Therapists expressed enthusiasm and professional satisfaction in seeing participants’ fatigue improve and the positive changes made in their lives because of their involvement in the LIFT randomized controlled trial.

I got a huge amount out of it, and the patients were great, I have to say, and I really … it was a great … when they were getting further and making progress and seeing differences themselves, it was a real boost, I think, for them and for me … that we were able to manage all this by ’phone and that they could see a difference in their lives. [T05 CBA]

I felt like had totally changed her life, and sort of socially, personally, professionally, just everything, she was like a different person. And that was really lovely, and I felt like I got to the end of it and thought, ‘I’ve really made a difference’, and I can see how the results of this would really show huge benefits. [T16 PEP]

#### Implementing the LIFT intervention in daily practice

The skills and tools acquired by therapists during LIFT training gave them greater confidence in the advice and support they offered to patients.

I don’t think I wasn’t saying the right thing before, I just was not as confident … we just referred them to the occupational therapist, whereas now I can just do the advice myself, and if I do have the time, I do explain how behaviours and thoughts can affect the way we act and how it’s all connected. [T01 CBA]

I’m not scared to get them doing more … [When] fatigue’s a big issue you think, ‘Oh, I don’t want them overdoing things’, whereas now I know that it helps … I’m not as reluctant … it’s certainly improved the overall management. [T12 PEP]

[LIFT] feels like the missing link to we what we were always doing. It’s made a massive difference; we were getting quite good results with the fatigue group, but I kind of felt like there was something missing, and I feel this has absolutely been it … listening, not trying to think of solutions and getting patients to come up with that themselves, and that’s what’s made the difference, without a doubt. [T07 CBA]

#### An intervention that still works with coronavirus disease

Many therapists shared ideas about how the interventions could be rolled out clinically. The only limitation to the intervention working remotely was securing private clinic space to make telephone or video calls to participants. To facilitate communication with patients, therapists suggested the option for video consultations, call headsets and using a data sharing app:

Certainly now, after COVID, there’s a lot more telephone work going on.… Theoretically, I guess, I could have done [LIFT] from home. [T09 CBA]

A face-to-face-type video chat might have been a bit more engaging. It was all ’phone calls, and you needed that first face to face, I think, session to get buy-in and build that rapport with your patient to get them to engage with the format, so probably something a bit more similar. [T12 PEP]

I do think video link is the way to go if patients are able to do that. Because it’s nice to see somebody and also the only drawback of the ’phone was I would have liked to have seen patients’ activity diaries. [T05 CBA].

## Discussion

Although rheumatology teams are increasingly aware that fatigue can be a challenging symptom for patients to manage, they have very few treatment options available to help [22]. These results have highlighted the benefits of health professionals receiving structured training and learning skills to support patients with fatigue. Although seen as awkward by some therapists, the use of role play during training allowed them the chance to practise their skills before undertaking sessions with patients. Role play encourages participation and the adoption of an identity, based on simulated scenarios, for educational purposes [23]. In modern medical education, role play is typically used to develop communication and critical thinking skills in clinical practice [24] and to enable health professionals to experience the imagined perspectives of the clinician and the patient [23]. In the present study, some patients showed a lack of engagement with the intervention. Although financial and time constraints on therapists’ time could potentially limit what can be offered to patients, there is potential for future therapist training to focus on engaging participants who are less willing to take part in the intervention. The benefits of skills training for rheumatology nurses, occupational therapists and physiotherapists to support patients with fatigue have been highlighted in self-management interventions for multiple sclerosis [25] and RA [26]. Health-care professionals who undertook an intensive self-management programme for patients with RA described how techniques such as motivational interviewing had seemed difficult initially, but had become easier with practice and had increased their professional confidence in supporting patients [27].

A further benefit of the LIFT randomized controlled trial was the clinical supervision that therapists could access. Supervision has been cited as a helpful element of other interventions, including delivery of a group fatigue intervention for RA patients by clinical teams [26]. Although the supervision in the present study was provided by experienced professionals to their less experienced colleagues, peer support might offer a more realistic and achievable model within NHS care that is worth pursuing in further studies. This might be particularly relevant in busy rheumatology departments [28]; for example, a rapid review of clinical supervision in the NHS found that peer supervision was perceived as a positive form of support. Helpful elements included supervisors’ self-disclosure regarding their own experiences, helping to normalize the supervisees’ experiences and encouraging them to share their viewpoints [28, 29]. For these benefits to influence patient care, it is vital that supervision be given regularly, with protected time for staff to take part in supervised practice [28].

Few LIFT therapists had previous experiences of delivering care over the telephone, but they were able to work effectively with remote delivery. Although some concerns have been raised regarding the potential disadvantages of telephone delivery, such as the inability to see facial expressions [30], and some patients have voiced scepticism [31], a recent systematic review comparing remotely delivered and face-to-face cognitive-behavioural therapy interventions found no significant effect on patient–therapist interactions [32]. Remotely delivered exercise interventions using videoconferencing were found to result in significantly greater 12-week weight loss compared with in-person or usual care arms [33] or a control group [34]. Telephone delivery offered several advantages to both participants and therapists. Although the present study was designed and delivered before the coronavirus disease 2019 (COVID-19) pandemic, therapists commented that most patient-facing rheumatology services had changed to remotely delivered consultations since March 2020.

Although the PEP and CBA interventions were perceived positively by therapists, they had several ideas for improvements before rolling them out to more NHS sites. These included the more widespread use of video consultations to facilitate communication, particularly when explaining exercises in the PEP intervention or sharing pictorial information, such as activity diaries, in the CBA intervention. Ideas for making data sharing between therapists and patients more streamlined were proposed, such as using a secure data-sharing app. In addition, future research could also explore the more cost-effective and practical means of delivering the intervention across a wider range of NHS sites and at lower cost. Future economic evaluation and analysis would be beneficial to evaluate whether the LIFT intervention offers cost savings compared with usual care.

### Strengths and limitations

A strength of this research is that therapists were contacted after they had finished delivering the interventions, giving them the opportunity to reflect on the whole process. Therapists in this study were based at six hospital sites across the UK and seemed very open to communicating about their experiences. This enabled exploration of a variety of viewpoints from therapists working in a range of clinical settings, serving different communities and with different local infrastructures that might impact their experiences.

A limitation is the small sample size of participants (*n* = 17) recruited to the qualitative evaluation sub-study. In addition, interviews with therapists after training and before delivery of LIFT might have provided more detail about their thoughts before starting the intervention.

### Conclusions

These findings support the value of skills training for rheumatology health professionals to deliver PEP and CBA fatigue-management interventions remotely. Therapists described many positives of the LIFT interventions, including professional satisfaction at seeing patients’ fatigue improve, increased confidence in supporting patients with fatigue, and the challenges and benefits of learning new skills. Valuable therapist-proposed ideas for positive changes to the LIFT interventions to improve the efficiency of delivery and information sharing have been proposed, which can be considered for wider roll out of the interventions in the future. Further research could also consider the most cost-effective and practical way to deliver the intervention across a wider range of study sites. These insights can inform service provision and clinical practice for remotely delivered support of rheumatology patients with fatigue.

## Supplementary data

Supplementary data are available at *Rheumatology Advances in Practice* online.

## Supplementary Material

rkac083_Supplementary_Data

## Data Availability

The data underlying this article cannot be shared publicly due to ethical reasons, to protect the privacy of individuals that participated in the study.

## References

[1] Belza BL , Henke CJ, Yelin EH, Epstein WV, Gilliss CL. Correlates of fatigue in older adults with rheumatoid arthritis. Nurs Res 1993;42:93–9. 8455994

[2] Pollard LC , Choy EH, Gonzalez J, Khoshaba B, Scott DL. Fatigue in rheumatoid arthritis reflects pain, not disease activity. Rheumatology 2006;45:885–9. 16449363 10.1093/rheumatology/kel021

[3] Hewlett S , Cockshott Z, Byron M et al Patients' perceptions of fatigue in rheumatoid arthritis: overwhelming, uncontrollable, ignored. Arthritis Care Res 2005;53:697–702. 10.1002/art.21450 16208668

[4] Tench CM , McCarthy J, McCurdie I, White PD, D'Cruz DP. Fatigue in systemic lupus erythematosus: a randomized controlled trial of exercise. Rheumatology 2003;42:1050–4. 12730519 10.1093/rheumatology/keg289

[5] Zonana-Nacach A , Roseman JM, McGwin G Jr et al Systemic lupus erythematosus in three ethnic groups. VI: Factors associated with fatigue within 5 years of criteria diagnosis. LUMINA Study Group. LUpus in MInority populations: NAture vs Nurture. Lupus 2000;9:101–9. 10787006 10.1191/096120300678828046

[6] Danoff-Burg S , Friedberg F. Unmet needs of patients with systemic lupus erythematosus. Behav Med 2009;35:5–13. 19297299 10.3200/BMED.35.1.5-13 PMC2687813

[7] Aissaoui N , Rostom S, Hakkou J et al Fatigue in patients with ankylosing spondylitis: prevalence and relationships with disease-specific variables, psychological status, and sleep disturbance. Rheumatol Int 2012;32:2117–24. 21516494 10.1007/s00296-011-1928-5

[8] Primdahl J , Hegelund A, Lorenzen AG et al The Experience of people with rheumatoid arthritis living with fatigue: a qualitative metasynthesis. BMJ Open 2019;9:e024338. 10.1136/bmjopen-2018-024338 PMC6475175 30898808

[9] Cramp F , Hewlett S, Almeida C et al Non-pharmacological interventions for fatigue in rheumatoid arthritis. Cochrane Database Syst Rev 2013;CD008322. 23975674 10.1002/14651858.CD008322.pub2 PMC11748118

[10] Main CJ , George SZ. Psychologically informed practice for management of low back pain: future directions in practice and research. Phys Ther 2011;91:820–4. 21451091 10.2522/ptj.20110060

[11] Dures E , Almeida C, Caesley J et al A survey of psychological support provision for people with inflammatory arthritis in secondary care in England. Musculoskelet Care 2014;12:173–81. 10.1002/msc.1071 PMC4282402 24753071

[12] Dures E , Hewlett S, Ambler N et al Rheumatology clinicians’ experiences of brief training and implementation of skills to support patient self-management. BMC Musculoskelet Disord 2014;15:108. 24678645 10.1186/1471-2474-15-108 PMC3978089

[13] Lamb SE , Lall R, Hansen Z et al; BeST Trial Group. A multicentred randomised controlled trial of a primary care-based cognitive behavioural programme for low back pain. The Back Skills Training (BeST) trial. Health Technol Assess 2010;14:1–253. iii–iv. 10.3310/hta14410 20807469

[14] Hewlett S , Almeida C, Ambler N et al; RAFT Study Group. Reducing arthritis fatigue impact: two-year randomised controlled trial of cognitive behavioural approaches by rheumatology teams (RAFT). Ann Rheum Dis 2019;78:465–72. 30793700 10.1136/annrheumdis-2018-214469 PMC6530078

[15] Martin KR , Bachmair EM, Aucott L et al Protocol for a multicentre randomised controlled parallel-group trial to compare the effectiveness of remotely delivered cognitive-behavioural and graded exercise interventions with usual care alone to lessen the impact of fatigue in inflammatory rheumatic diseases (LIFT). BMJ Open 2019;9:e026793. 10.1136/bmjopen-2018-026793 PMC6359876 30705244

[16] Bachmair E-M , Martin K, Aucott L et al Remotely delivered cognitive behavioural and personalised exercise interventions for fatigue severity and impact in inflammatory rheumatic diseases (LIFT): a multicentre, randomised, controlled, open-label, parallel-group trial. Lancet Rheumatol 2022;4:e534–45. 10.1016/S2665-9913(22)00156-4 PMC9646481 36388001

[17] DiCicco-Bloom B , Crabtree BF. The qualitative research interview. Med Educ 2006;40:314–21. 16573666 10.1111/j.1365-2929.2006.02418.x

[18] Mealer M , Jones J. Methodological and ethical issues related to qualitative telephone interviews on sensitive topics. Nurse Res 2014;24:32–7. 10.7748/nr2014.03.21.4.32.e1229 24673351

[19] QSR International Pty Ltd. NVivo (Version 12). 2018. https://www.qsrinternational.com/nvivo-qualitative-data-analysis-software/home.

[20] Braun V , Clarke V. Using thematic analysis in psychology. Qual Res Psychol 2006;3:77–101.

[21] Saunders B , Sim J, Kingstone T et al Saturation in qualitative research: exploring its conceptualization and operationalization. Qual Quant 2018;52:1893–907. 29937585 10.1007/s11135-017-0574-8 PMC5993836

[22] Repping-Wuts H , Hewlett S, van Riel P, van Achterberg T. Fatigue in patients with rheumatoid arthritis: British and Dutch nurses' knowledge, attitudes and management. J Adv Nurs 2009;65:901–11. 19243466 10.1111/j.1365-2648.2008.04904.x

[23] Bosse HM , Nickel M, Huwendiek S et al Peer role-play and standardised patients in communication training: a comparative study on the student perspective on acceptability, realism, and perceived effect. BMC Med Educ 2010;10:27. 20353612 10.1186/1472-6920-10-27 PMC2853557

[24] Latif R , Mumtaz S, Mumtaz R, Hussain A. A comparison of debate and role play in enhancing critical thinking and communication skills of medical students during problem based learning. Biochem Mol Biol Educ 2018;46:336–42. 29668075 10.1002/bmb.21124

[25] Peters S , Wilkinson A, Mulligan H. Views of healthcare professionals on training for and delivery of a fatigue self-management program for persons with multiple sclerosis. Disability and Rehabilitation 2019;41:2792–8. 29911446 10.1080/09638288.2018.1478993

[26] Dures E , Rooke C, Hammond A, Hewlett S. Training and delivery of a novel fatigue intervention: a qualitative study of rheumatology health-care professionals’ experiences. Rheumatol Adv Pract 2019;3:rkz032. 31559382 10.1093/rap/rkz032 PMC6755488

[27] Prothero L , Sturt J, de Souza S et al; TITRATE Programme Investigators. Intensive management for moderate rheumatoid arthritis: a qualitative study of patients’ and practitioners’ views. BMC Rheumatol 2019;3:12. 30976751 10.1186/s41927-019-0057-8 PMC6437952

[28] Rothwell C , Kehoe A, Farook S, Illing J. The characteristics of effective clinical and peer supervision in the workplace: a rapid evidence review. Newcastle upon Tyne: Newcastle University, 2019:69.

[29] MacLaren J-A. Supporting nurse mentor development: an exploration of developmental constellations in nursing mentorship practice. Nurse Educ Pract 2018;28:66–75. 28965020 10.1016/j.nepr.2017.09.014

[30] Turner J , Brown JC, Carpenter DT. Telephone-based CBT and the therapeutic relationship: the views and experiences of IAPT practitioners in a low-intensity service. J Psychiatr Ment Health Nurs 2018;25:285–96. 29117458 10.1111/jpm.12440

[31] Haller E , Besson N, Watzke B. “Unrigging the support wheels” - a qualitative study on patients’ experiences with and perspectives on low-intensity CBT. BMC Health Serv Res 2019;19:686. 31597555 10.1186/s12913-019-4495-1 PMC6784338

[32] Irvine A , Drew P, Bower P et al Are there interactional differences between telephone and face-to-face psychological therapy? A systematic review of comparative studies. J Affect Disord 2020;265:120–31. 32090733 10.1016/j.jad.2020.01.057 PMC7049904

[33] Johnson KE , Alencar MK, Coakley KE et al Telemedicine-based health coaching is effective for inducing weight loss and improving metabolic markers. Telemed J E-Health 2019;25:85–92. 29847222 10.1089/tmj.2018.0002 PMC6384514

[34] Alencar MK , Johnson K, Mullur R et al The efficacy of a telemedicine-based weight loss program with video conference health coaching support. J Telemed Telecare 2019;25:151–7. 29199544 10.1177/1357633X17745471

