# Supplementary material for: Therapists’ experiences of remotely delivering cognitive-behavioural or graded-exercise interventions for fatigue: a qualitative evaluation
Source: Rheumatol Adv Pract. 2022 Oct 17;6(3):rkac083. doi: 10.1093/rap/rkac083 (PMC9629972; doi:10.1093/rap/rkac083)
Supplement: rkac083_Supplementary_Data [file rkac083_supplementary_data.zip › 22-020 Supplementary Data S2. LIFT Study Team.docx]

**Supplementary Data S2. LIFT Study Team**

The authors would like to thank the members of the LIFT Study Team: Lorna Aucott (Health Service Research Unit), Kathryn Martin (University of Glasgow, Glasgow, UK; Aberdeen Centre for Arthritis and Musculoskeletal Health), Neeraj Dhaun (British Heart Foundation Centre of Research Excellence, Centre for Cardiovascular Science, The Queen’s Medical Research Institute), Richard Emsley (Department of Biostatistics and Health Informatics, Institute of Psychiatry, Psychology and Neuroscience, King’s College London, London, UK), Elizabeth Kidd (Department of Rheumatology, Freeman’s Hospital, The Newcastle upon Tyne Hospitals NHS Foundation Trust, Newcastle upon Tyne, UK), Vinod Kumar (Department of Rheumatology, Ninewells, Hospital, NHS Tayside, Dundee, UK), Graeme MacLennan (Centre of Healthcare and Randomised Trials (CHaRT), Health Service Research Unit), Paul McNamee (Health Economics Research Unit, University of Aberdeen, Aberdeen, UK), John Norrie (Edinburgh Clinical Trials Unit, University of Edinburgh, Edinburgh, UK), Jon Packham (Physiotherapy and Paramedicine, Haywood Rheumatology Centre, Stoke-on-Trent, UK), Stuart H Ralston (Rheumatology and Bone Disease), Stefan Siebert (Institute of Infection, Immunity and Inflammation, University of Glasgow, Glasgow, UK), Amy Nicol, Karen Norris, Sandra Mann, Lorna Van Lierop, Eli Gomez, Fiona McCurdy, Valerie Findlay, Neil Hastie, Eunice Morgan, Roselyn Emmanuel, Daniel Whibley, Aimee Urquart, Laura MacPerson (NHS Grampian, UK); Janice Rowland, Gwen Kiddie, Debbie Pankhurst, Paul Johnstone, Hilary Nicholson, Angela Dunsmore, Alison Knight, John Ellis, Callum Maclean, Linda Crighton, Cameron Shearer (NHS Tayside, UK); Judy Coyle, Susan Begg, Lyndsey Ackerman, Jill Carnevale, Samantha Arbuthnot, Helen Watters, Dervil Dockrell, Debbie Hamilton (NHS Lothian, UK); Dario Salutous, Susanne Cathcart, Dominic Rimmer, Emma Hughes, Juliet Harvey, Mairi Gillies, Susan Webster, Leeanne Milne, Gary Semple, Katharine Duffy, Lynne Turner, John Alexander, June Innes, Charlotte Clark, Christine Meek, Elizabeth McKenna (NHS Greater Glasgow and Clyde, UK); Christine Routledge, Helain Hinchcliffe-Hume, Emmanuella Traianos, Beth Dibnah, David Storey, Gemma O’Callaghan, Jenny Yael Baron, Sally Hunt (Newcastle upon Tyne NHS Trust); Natalie Wheat, Pam Smith, Elizabeth Ann Barcroft, Amy Thompson, Johanne Tomlinson (Haywood Hospital, Stoke on Trent, UK); Jill Barber, Gladys MacPerson (University of Aberdeen, UK); Peter White (Queen Mary University of London, UK); Sarah Hewlett (University of the West of England, Bristol, UK).
