# Supplementary material for: Therapists’ experiences of remotely delivering cognitive-behavioural or graded-exercise interventions for fatigue: a qualitative evaluation
Source: Rheumatol Adv Pract. 2022 Oct 17;6(3):rkac083. doi: 10.1093/rap/rkac083 (PMC9629972; doi:10.1093/rap/rkac083)
Supplement: rkac083_Supplementary_Data [file rkac083_supplementary_data.zip › Supplementary Data S1. LIFT_Therapists.docx]

**LIFT Tutor Interview Guide: CBA**

**Introduction**

This is an opportunity to discuss your experience of LIFT. Hearing about your experiences will help us to understand the practicalities, challenges and benefits of training nurses & allied health professionals to deliver the programme. As well as telling us about this research trial, your views and ideas will inform how LIFT might be rolled out in the future.

**Prior to LIFT**

Please tell me about:

- Your reason(s) for deciding to take part in LIFT
- Any previous experience of using cognitive-behavioural (CB) techniques
- Any other relevant experience (e.g. training in motivational interviewing)

**Training**

We would like hear your thoughts on the 3 day training that you did:

- The content (e.g. CB theory, manual content, key messages)
- The structure (e.g. role play, demonstrations, a single block of 3 days)
- Your experience (e.g. was it what you expected? More/less challenging?)
- Would you suggest any changes (e.g. more/less skills practice, theory, or focus on specific sessions)?
- How did you feel about the idea of delivering LIFT after completing the 3 day training (your confidence, motivation, the perceived importance of the programme)?

**Delivery**

We would like hear your thoughts on delivering LIFT:

- Practical challenges (e.g. time to practice and prepare, using telephones internet based calls)
- Personal/professional challenges (e.g. learning new skills, learning new information, using a manual)
- Were there particular sessions or aspects of LIFT that you found problematic or did not like?
- Were there particular sessions or aspects of LIFT that you liked more than others?
- Clinical supervision
  - Was this a helpful part of the process? If so, how?
  - Were there any particular issues that you sought support for?
  - Did the nature of clinical supervision change over time?
- Did delivery of LIFT change each time? If so, how?

**Impact of wider clinical practice**

- Has taking part in LIFT had any impact on your wider clinical practice? If so, can you give me some examples (e.g. materials or techniques that you find useful)
- Do you perceive any benefits to participants? / Do you perceive any drawbacks for participants?
- Do you perceive any benefits for your professional development? / Do you perceive any drawbacks for your professional development?

**In the future**

Thinking about how we might roll out LIFT in the future:

- Would you recommend changes to the manual? If so, can you describe them?

**Close**

Are there any other aspects of your experience or thoughts about LIFT in the future that you would like to tell us about?

Thank you for your time

**LIFT Tutor Interview Guide: PEP**

**Introduction**

This is an opportunity to discuss your experience of LIFT. Hearing about your experiences will help us to understand the practicalities, challenges and benefits of training physiotherapists & allied health professionals to deliver the programme. As well as telling us about this research trial, your views and ideas will inform how LIFT might be rolled out in the future.

**Prior to LIFT**

Please tell me about:

- Your reason(s) for deciding to take part in LIFT
- Any other relevant experience

**Training**

We would like hear your thoughts on the 3 day training that you did:

- The content (e.g. fatigue theory, manual content, key messages)
- The structure (e.g. demonstrations, a single block of 3 days)
- Your experience (e.g. was it what you expected? More/less challenging?)
- Would you suggest any changes (e.g. more/less skills practice, theory, or focus on specific sessions)?
- How did you feel about the idea of delivering LIFT after completing the 3 day training (your confidence, motivation, the perceived importance of the programme)?

**Delivery**

We would like hear your thoughts on delivering LIFT:

- Practical challenges (e.g. time to practice and prepare, initial face-to-face assessment and using telephones internet-based calls for remaining sessions)
- Personal/professional challenges (e.g. learning new skills, learning new information, using a manual)
- Were there particular sessions or aspects of LIFT that you found problematic or did not like?
- Were there particular sessions or aspects of LIFT that you liked more than others?
- Clinical supervision
  - Was this a helpful part of the process? If so, how?
  - Were there any particular issues that you sought support for?
  - Did the nature of clinical supervision change over time?
- Did delivery of LIFT change each time? If so, how?

**Impact of wider clinical practice**

- Has taking part in LIFT had any impact on your wider clinical practice? If so, can you give me some examples (e.g. materials or techniques that you find useful)
- Do you perceive any benefits to participants? / Do you perceive any drawbacks for participants?
- Do you perceive any benefits for your professional development? / Do you perceive any drawbacks for your professional development?

**In the future**

Thinking about how we might roll out LIFT in the future:

- Would you recommend changes to the manual? If so, can you describe them?

**Close**

Are there any other aspects of your experience or thoughts about LIFT in the future that you would like to tell us about?

Thank you for your time
